# On the Comparative Analysis of Different Phase Coexistences in Mesoporous Materials

**DOI:** 10.3390/ma15072350

**Published:** 2022-03-22

**Authors:** Henry R. N. B. Enninful, Dirk Enke, Rustem Valiullin

**Affiliations:** 1 Felix Bloch Institute for Solid State Physics, Leipzig University, 04103 Leipzig, Germany; 2 Institute for Technical Chemistry, Leipzig University, 04103 Leipzig, Germany; dirk.enke@uni-leipzig.de (D.E.); valiullin@uni-leipzig.de (R.V.)

**Keywords:** porous solids, phase equilibria, nitrogen sorption, NMR cryoporometry, thermoporometry, mercury porosimetry

## Abstract

Alterations of fluid phase transitions in porous materials are conventionally employed for the characterization of mesoporous solids. In the first approximation, this may be based on the application of the Kelvin equation for gas–liquid and the Gibbs–Thomson equation for solid–liquid phase equilibria for obtaining pore size distributions. Herein, we provide a comparative analysis of different phase coexistences measured in mesoporous silica solids with different pore sizes and morphology. Instead of comparing the resulting pore size distributions, we rather compare the transitions directly by using a common coordinate for varying the experiment’s thermodynamic parameters based on the two equations mentioned. Both phase transitions in these coordinates produce comparable results for mesoporous solids of relatively large pore sizes. In contrast, marked differences are found for materials with smaller pore sizes. This illuminates the fact that, with reducing confinement sizes, thermodynamic fluctuations become increasingly important and different for different equilibria considered. In addition, we show that in the coordinate used for analysis, mercury intrusion matches perfectly with desorption and freezing transitions.

## 1. Introduction

Alterations of fluid phase equilibria in porous materials are important markers to understand fluid behavior in confined pore spaces [1,2]. In reservoir rocks, for example, they predict the nature and characteristics of reservoirs and help devise suitable methods to enhance recovery efficiencies of underground resources [3,4]. Under varying temperatures, water formed in crevices and cracks of aerosol particles creates the congenial conditions for ice formation in clouds [5,6]. Furthermore, such changes in fluid behavior provide essential details about the morphology and texture of the confinements as a necessary step towards their optimization for various industrial applications, such as catalysis, energy storage, molecular separations, and drug delivery [7,8,9,10].

Gas–liquid and solid–liquid phase equilibria are the two most common phenomena employed in the textural characterization of mesoporous materials [11,12]. When a gas under low pressure is brought into contact with a mesoporous material, the gas molecules first adsorb onto the pore walls until sufficient coverage has been obtained. An increase in gas pressure leads to the creation of multiple layers of adsorbed gas film. At a higher pressure, thermal fluctuations of the gas–liquid interfaces may trigger the creation of a liquid bridge. The entire pore is then filled at that pressure, a phenomenon known as capillary condensation. On desorption, gas from the surrounding gas bath invades the pore at the equilibrium gas pressure of the pore, i.e., such pressure that the receding meniscus does not change the total free energy of the system. This provides useful information about the pore size. In its approximate form, the meniscus radius, r, can be described by the classical Kelvin equation given by [13]

(1) $$ln\frac{p}{p_{0}}=\frac{2\gamma_{gl}V_{m}}{rRT}$$

where *p* is the actual vapor pressure, *p*_0_ saturated vapor pressure, *γ_gl_* vapor–liquid interfacial energy, *V_m_* molar volume, *R* universal gas constant, and *T* is the temperature of the gas. When the adsorbed film thickness is added, the pore size is obtained.

Analogous to gas sorption is thermoporometry (or its special realization NMR Cryoporometry), where a pore filled with frozen liquid, for example, ice, eventually melts upon heating and, thus, yields information about the pore structure [14]. As a means to lower the free energy of the system, the system favors the existence of a liquid film between the frozen core and pore wall, commonly known as the non-frozen layer (NFL) or pre-molten layer. Upon heating, the NFL thickness increases until the thermodynamic barrier for liquid formation is overcome by thermal fluctuations, resulting in pore melting. Upon temperature reduction, the surrounding bulk frozen phase with a bulk melting temperature, *T*_0_, forms a hemispherical cap at the pore mouth–bulk phase interface. The elimination of thermodynamic barriers at the pore equilibrium transition temperature leads to intrusion of the ice front into the pore filled with molten liquid of molar volume, *V_m_* [15]. At this equilibrium temperature, *T*, the entire pore freezes, excluding a few monolayers of NFL between the frozen core of size, *r*, and the pore wall. With enthalpy of fusion, Δ*H_f_*, and solid–liquid interfacial energy, *γ_sl_*, the temperature suppression on the formation of the solid core is generally captured by the well-known Gibbs–Thomson equation as [16]

(2) $$\frac{T-T_{0}}{T_{0}}=\frac{2\gamma_{sl}V_{m}}{r\Delta H_{f}}$$

It has long been recognized that these two phenomena share common physics and, hence, attempts to interrelate freezing–melting and desorption–adsorption have been made [17]. Indeed, Equations (1) and (2) have close similarities in such a way that unifying them through a common framework for comparing nitrogen desorption and freezing in materials of at least a mean pore size of 5 nm and a narrow pore width distribution show very good agreement [17,18]. The usefulness of such comparative analysis is easily seen by referring to a wealth of new information one may obtain using different gases for gas sorption studies [19,20]. 

The influence of pore size and temperature on the hysteresis width of adsorption isotherms has been well documented in the literature for various mesoporous materials such as MCM-41 and SBA-15 [21]. At a given temperature, sorption hysteresis vanishes below a critical pore size. Additionally, for a given pore size, hysteresis disappears above a certain critical temperature [22,23]. The thermodynamic properties of the adsorbate are responsible for both observed phenomena.

For a more direct way to view phase equilibria and hysteresis by nitrogen sorption at 77 K and cryoporometry with water in a series of mesoporous materials of different pore sizes and morphologies, we visualize the transitions in a unified coordinate system similar to that suggested by Denoyel et al. [18]. In this way, we are able to compare phase equilibria in both processes and obtain a deeper understanding of phase behavior due to pore size and morphology. Particularly, it helps to reveal effects beyond the validity of the Kelvin and Gibbs–Thomson equations. Furthermore, in conjunction with data from mercury porosimetry, we show a more general perspective of the approach suggested.

## 2. Materials and Methods

To analyze the two different phase transitions, data of nitrogen adsorption and NMR cryoporometry of water measured in some mesoporous solids of varying morphology were used. The materials include SBA-15 and MCM-41 synthesized and used in our previous studies [24], VYCOR from Corning Inc., and controlled porous glass (CPG) from Fluka Chemie AG. Nitrogen sorption at 77 K and NMR cryoporometry of water on heating and cooling cycles over several temperatures were employed for this study. Detailed experimental protocols on the methods used have been described elsewhere [25]. The raw data from the measurements without corrections for the adsorbed film thickness and NFLs have been used. NMR cryoporometry experiments were typically performed using the Hahn echo and Carr–Purcell–Meiboom–Gill (CPMG) pulse sequences including an interpulse delay, which acts as a filter to eliminate signals from the ice phase and part of the NFLs.

Additionally, measurements of mercury porosimetry have been made on the large- pore-sized CPG material. Prior to the mercury intrusion experiments, the sample was outgassed at 0.2 mbar for 10 min at ambient temperature prior to filling the dilatometer with mercury. A total of 55 mg of the CPG sample were obtained and mercury intrusion measurements were performed with a PASCAL 440 porosimeter from ThermoScientific/POROTEC within the range from ambient pressure up to 4000 bar. The mercury surface tension was assumed to be 0.48 N/m and its contact angle set to 140° [26].

## 3. Results and Discussion

### 3.1. A Unified Coordinate System for Analysis

The Kelvin equation, Equation (1), relates the size of the gaseous domains, *r*, with the relative gas pressure, $ln\frac{p}{p_{0}}$. Similarly, the Gibbs–Thomson equation, Equation (2), relates the size of the in-pore frozen core formed on freezing, *r*, with the ratio of temperature suppression to bulk temperature, $\frac{T-T_{0}}{T_{0}}$. For many liquids, the melting temperature suppression in mesopores, $T-T_{0}$, is relatively small compared to the bulk transition temperature, $T_{0}$. Hence,

(3) $$ln\frac{T}{T_{0}}\approx \frac{T-T_{0}}{T_{0}}$$

By the approximation in Equation (3), Equations (1) and (2) become mathematically similar and allows the introduction of a general coordinate for both phenomena. Indeed, on considering the curvature, $\frac{2}{r}$, as a common parameter for both methods, the general coordinate, x, becomes

(4) $$x=A^{-1}ln\left(z\right)$$

where *A* and *z* are respectively equal to $\frac{\gamma_{gl}V_{m}}{RT}$ and $\frac{p}{p_{0}}$ for the Kelvin equation and $\frac{\gamma_{sl}V_{m}}{\Delta H_{f}}$ and $\frac{T}{T_{0}}$ for the Gibbs–Thomson equation can be suggested. It is expected that under the conditions where both Kelvin and Gibbs–Thomson equations accurately describe the phase transitions in the pore spaces, the courses of the phase transitions plotted using the general coordinate should overlap.

### 3.2. Experimental Visualizations

Following the creation of the general coordinate axis, we can now conduct the comparative analysis of data from nitrogen sorption at 77 K and NMR cryoporometry of water measured in the selected mesoporous materials.

**Figure 1:**
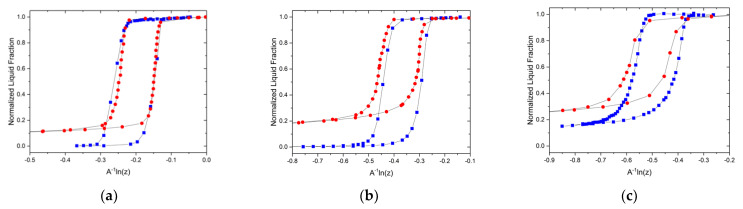
Comparison of nitrogen sorption isotherms (red circles) and freezing/melting transitions for water (blue squares) in (**a**) controlled porous glass of mean pore size ≈ 12.8 nm; (**b**) controlled porous glass of mean pore size ≈ 10.5 nm and (**c**) SBA-15 of mean pore size ≈ 8 nm. Figure (**c**) has been reproduced with permission from ACS Publications [27].

Figure 1 shows the results obtained for nitrogen sorption and NMR cryoporometry on CPG and SBA-15 mesoporous materials. It shows the normalized liquid fractions in the pore spaces. In materials with relatively large pore sizes (Figure 1a), the fraction of the adsorbed nitrogen film thickness and NFL is small compared to the pore size. Hence, their variations with temperature or pressure do not appreciably affect the notably steeper changes in the liquid fractions due to phase transitions in the pores. This renders the Kelvin and Gibbs–Thomson equations accurate in describing the transitions. It explains, in part, the perfect overlap of the transitions measured for both equilibria. With decreasing pore size, as seen in Figure 1b,c, the influence of the adsorbed film thickness becomes visible and reveals slight deviations from a perfect overlap. Irrespective of these deviations, it is seen, however, that the ranges of x where the transitions occur coincide. If the variation in the film or NFL thicknesses is properly accounted for, then the overlap will be perfect. This, however, is out of the scope of this short communication.

**Figure 2:**
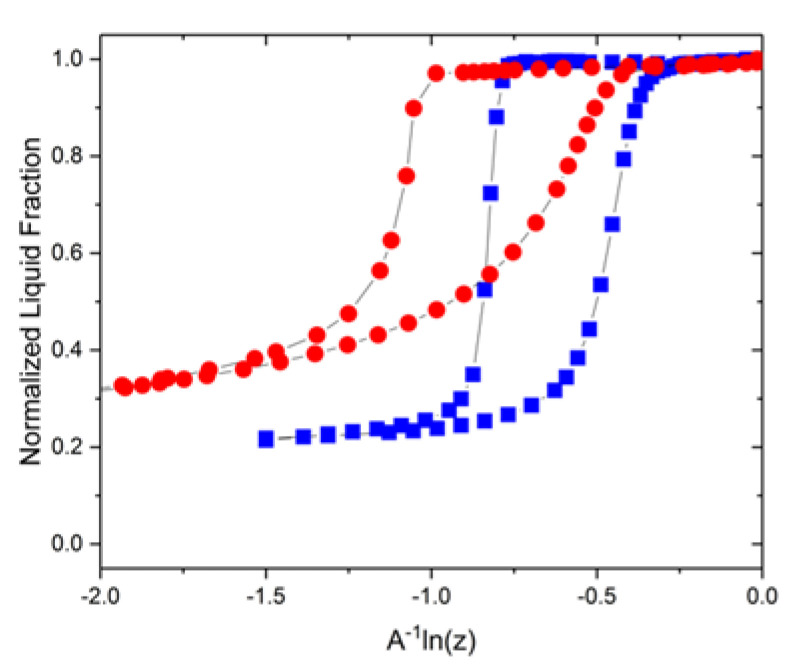
Comparison of nitrogen sorption isotherms (red circles) and NMR cryoporometry transitions (blue squares) of water in VYCOR porous glass.

In the plot obtained for the VYCOR porous glass, the adsorption isotherm shows a reasonably good agreement with the melting transition. Note that even though the variations in the adsorbed film and NFL thicknesses contribute substantially to the normalized transition curves, the capillary–condensation and melting transitions occur in the identical ranges of the common x-coordinate. On the other hand, a notable divergence is observed between the desorption isotherm and freezing transition, clearly pointing out that the phase transitions occur at different values of x in Equation (4). From Figure 2, desorption occurs at a lower value of x, resulting in a larger hysteresis for the sorption isotherm. To rationalize this, we consider the property of the surface roughness of phase growth processes. This property, accounted for by the so-called Jackson α-factor [28], describes the influence of thermal fluctuations on surface roughening of growth fronts. Defined as

(5) $$\alpha =\frac{\eta }{Z}\frac{L}{RT_{0}}$$

it is determined by the balance between entropic and enthalpic contributions to the free energy of the growing crystal. In Equation (5), *η* is the number of atomic bonds in a molecular layer, *Z* is the crystal coordination number, *L* represents the enthalpy of fusion of ice, *R* the universal gas constant, and *T*_0_ is the bulk melting transition temperature for ice.

The higher the α-factor, the weaker the thermal fluctuations, resulting in layer-by-layer phase growth. A lower α-factor, however, produces more dendritic growth at growth fronts [29]. In other words, the alpha factor determines amplitudes of the positional fluctuations of the solid–liquid front and they have been shown to affect the phase transition behaviors in mesoporous solids [30]. Though the α-factor is notably applied for the description of solid–liquid interface types, it may be extended to gas–liquid interfaces as a reasonable approximation. Here, an analog of the alpha factor is

(6) $$\alpha =\frac{\eta }{Z}\frac{\Delta H}{RT_{B}}$$

where Δ*H* is the enthalpy of vaporization and *T_B_* is the boiling temperature of the liquid. Assuming the molecular structure of ice and liquid nitrogen does not vary significantly, $\frac{\eta }{Z}$ for ice and liquid nitrogen vary marginally. Employing the constants present in Equations (5) and (6) and shown in Table 1, the values of α-factor for ice and its analog for the gas–liquid system become α_ice_ ≈ 2.6 and α_nitrogen_ ≈ 4.4, respectively. This implies that the interface fluctuations are stronger for the case of the propagating ice rather than the propagating gaseous phase.

**Figure 3:**
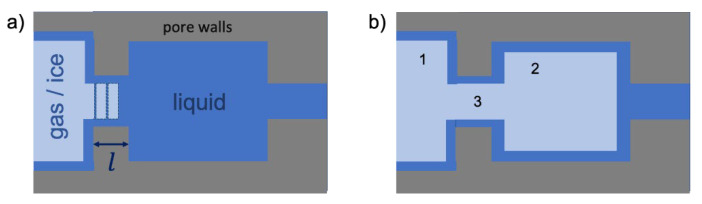
A schematic showing a part of a pore network in which a pore 1 containing either gas or ice phase is connected to a pore 2 with the capillary-condensed liquid via a smaller neck 3 with a length l. In (**a**), the positional gas/ice–liquid interface fluctuations in the neck are indicated. If their amplitude exceeds the neck length l, then the system jumps from a higher energy (**a**) to a lower energy (**b**) stable configuration.

To rationalize the observed divergence of the desorption isotherm from the freezing transition, let us consider the schematic drawing in Figure 3.

The figure shows schematically a particular configuration of an invaded gas or ice attained for a certain x (as determined by pressure or temperature). The situation exemplified by Figure 3a corresponds with a higher free energy, while that in Figure 3b with a lower one. The occurrence of the necks results in the respective barrier in the free energy that needs to be overcome to lower the energy. If the neck length is relatively long, then this process is delayed until a pressure or temperature when the gas or ice phase will invade the neck without the change in the free energy (equilibrium transition). If the length is, however, relatively short, then this can occur at the lower pressures and temperatures facilitated by thermal fluctuations in the axial direction. The efficiency of this process is determined by the strength of the positional interface fluctuations. As discussed earlier, for the water/ice interface at 273 K, these fluctuations are stronger than for the nitrogen gas/condensed liquid interface at 77 K. Hence, the ice invasion can happen at higher values of x than gas invasion. In VYCOR porous glass, where a large number of pore necks with relatively short lengths are encountered, the effect of thermal fluctuations become more pronounced. Consequently, this leads to the pattern revealed in Figure 2. Thermal fluctuations become significant in small pores (~5 nm) and increase gradually in smaller pores until very small pore sizes where no transition can occur [24].

**Figure 4:**
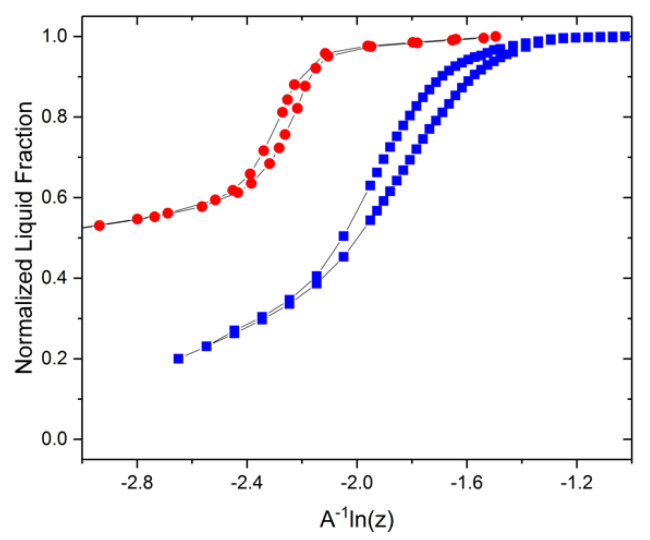
Comparison of nitrogen sorption isotherms (red circles) and NMR cryoporometry transitions (blue squares) data of MCM-41 with mean pore size ≈ 4 nm.

In MCM-41, clear distinctions in the nitrogen isotherm and NMR cryoporometry transitions are seen as shown in Figure 4. In pores of such small sizes, the classical Kelvin and/or Gibbs–Thomson equations break down and the figure confirms this. In this case, very strong confinements can give rise to a substantial effect of thermal fluctuations, but rather in the radial direction. In line with the discussion presented earlier revealing stronger fluctuations for the ice/water interface, the same trend is seen in Figure 4 where the cryoporometry transition occurs at substantially larger values of x.

### 3.3. Adjustment for Mercury Porosimetry

To extend the comparative analysis of phase transitions to include mercury porosimetry, the coordinate axis of the plot is adjusted to allow for analysis with mercury intrusion data. Mercury porosimetry employs the curvature effect due to the non-wetting property of mercury on most surfaces to characterize pore spaces. Given the surface tension, *γ* = 0.48 N/m, and mean contact angle, *θ* = 140°, between mercury and the pore surface, the applied external hydrostatic pressure, *P_Hg_*, needed to ensure the intrusion of mercury into a pore of size, *r_p_*, is defined as

(7) $$P_{Hg}=-\frac{2\gamma cos\theta }{r_{p}}$$

**Figure 5:**
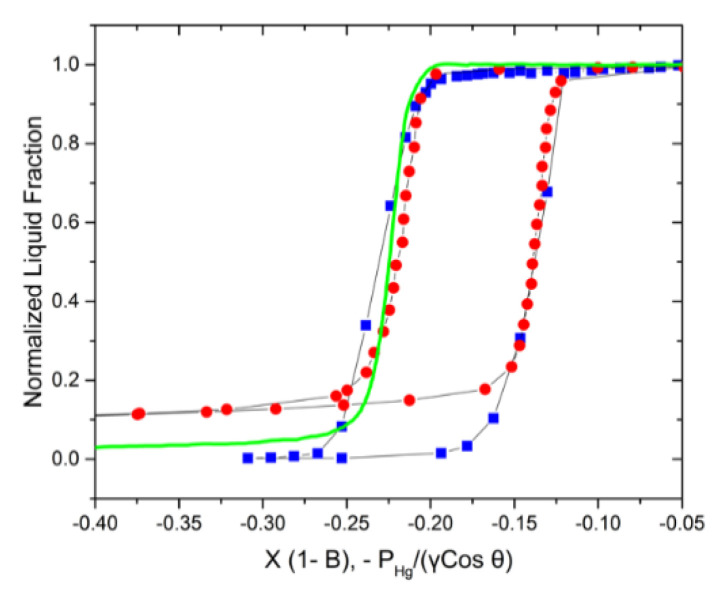
Comparison of nitrogen sorption isotherms (red circles), NMR water cryoporometry transitions (blue squares), and mercury intrusion (green line) data of a controlled porous glass of mean pore size ≈ 12.8 nm.

The pore curvature, $\frac{2}{r_{p}}$, therefore becomes equal to $-\frac{P_{Hg}}{\gamma cos\theta }$. On intrusion, the creation of the hemispherical curvature of the intruding mercury front closely resembles the one on gas invasion and ice intrusion into a pore upon adsorption and freezing, respectively. Noting that Equation (7) is based on the pore size, while Equations (1) and (2) for gas sorption and thermoporometry/NMR cryoporometry operate with the critical core sizes of the intruding phases, corrections for the adsorbed film thickness and NFLs need to be made for an accurate comparison. Recalling the coordinate axis of the plot of the phase equilibria, x = *A***^−^**^1^ln(z), the adjusted x-axis, therefore, becomes x (1 – B), where B = $\frac{\tau }{\left(\frac{-2}{r}\right)+\tau }$ if *τ* is the thickness of the adsorbed gas film or the NFLs and *r* is the radius of the invaded gas meniscus or the frozen core. The resulting plot is shown in Figure 5. It reveals a perfect agreement between the results obtained using the three different experimental approaches.

## 4. Conclusions

A unified framework, similar to that developed by Denoyel et al. [18], has allowed for a comparative analysis of the gas–liquid and solid–liquid phase equilibria in a set of mesoporous materials with variable pore sizes. This allowed us to show the similarities in hysteresis shapes and provided key insights into the differences in phase equilibria in mesoporous materials with smaller pore sizes and complex morphologies. In large mesopore size materials, in which the Kelvin and Gibbs–Thomson equation quite accurately capture the behavior, a perfect overlap between the sorption isotherm and cryoporometry phase transitions is obtained. In smaller pores, the overlap of transitions from both equilibria reveals the increasing influence of thermodynamic fluctuations giving rise to discrepancies between selected transition branches or both branches. Comparison of the phase transitions to mercury porosimetry show excellent agreement for the selected material. This framework may be extended to understand phase coexistence in spherical pores [31].

## Figures and Tables

**Table 1:** A table showing the values of the constants of ice and liquid nitrogen employed in the determination of the α-factor.

Material	L/ΔH $\left(\mathrm{kJ}\cdot {\mathrm{mol}}^{-1}\right)$	$T_{0}/T_{B}\left(K^{-1}\right)$
Ice	6.0	273
Nitrogen	2.8	77

## Data Availability

Data are contained within the manuscript.
